# Multicolour Multilevel STED nanoscopy of Actin/Spectrin Organization at Synapses

**DOI:** 10.1038/srep26725

**Published:** 2016-05-25

**Authors:** Sven C. Sidenstein, Elisa D’Este, Marvin J. Böhm, Johann G. Danzl, Vladimir N. Belov, Stefan W. Hell

**Affiliations:** 1 Max Planck Institute for Biophysical Chemistry, Department of NanoBiophotonics, Am Fassberg 11, 37077 Göttingen, Germany

## Abstract

Superresolution fluorescence microscopy of multiple fluorophores still requires development. Here we present simultaneous three-colour stimulated emission depletion (STED) nanoscopy relying on a single STED beam at 620 nm. Toggling the STED beam between two or more power levels (“multilevelSTED”) optimizes resolution and contrast in all colour channels, which are intrinsically co-aligned and well separated. Three-colour recording is demonstrated by imaging the nanoscale cytoskeletal organization in cultured hippocampal neurons. The down to ~35 nm resolution identified periodic actin/betaII spectrin lattices along dendrites and spines; however, at presynaptic and postsynaptic sites, these patterns were found to be absent. Both our multicolour scheme and the 620 nm STED line should be attractive for routine STED microscopy applications.

Far-field optical superresolution techniques such as stimulated emission depletion (STED) nanoscopy are on track to become standard methods for molecule-specific imaging in the life sciences and beyond^1^,^2^. At present, however, simultaneous recordings of more than two fluorophore species still require complex setups and/or analysis algorithms^3^,^4^. Here, we utilize a pulsed fibre laser emitting at 620 nm as the STED light source and demonstrate its versatility in a novel three-colour STED microscopy scheme.

Although STED has been implemented with laser lines in basically any part of the visible spectrum, the most widely established laser lines are 590 nm and 775 nm for de-exciting green or yellow fluorescent proteins (GFP/YFP) and organic synthetic dyes, respectively. There are several reasons for trying out a 620 nm laser line for STED. First, this wavelength is situated in the red-orange part of the visible spectrum, where water absorption is lowest and one can still expect to accommodate GFP/YFP. Second, moving the STED wavelength from the yellow-orange towards the red-orange spectrum should enable dyes with fluorescence peaking around 550 nm, which cannot be de-excited at ~590 nm without pronounced anti-Stokes excitation. Thus, a broad array of synthetic and genetically encoded markers comes to the fore.

In fact, STED nanoscopy of Atto532 (λ_abs_ 532 nm, λ_em_ 553 nm) was already demonstrated with STED at 615 nm^5^ and 620 nm^6^, albeit by using complicated and expensive femtosecond modelocked laser systems that provided pulses of unfavourable subpicosecond duration requiring substantial stretching. In contrast, the compact STED laser source harnessed in this study delivers pulses of ~600 ps width at 40 MHz repetition rate. It provides a good compromise between reduced fluorophore bleaching (due to longer pulses, minimizing non-linear photon absorption) and still short pixel dwell times of few tens of microseconds^3^,^7^,^8^.

Here, we developed a new imaging platform based on the 620 nm laser source for multicolour STED nanoscopy in living and fixed samples. Switching the STED power between different values (multilevelSTED) enables imaging with no compromise in contrast and resolution for all simultaneously recorded channels. The intrinsically co-aligned multicolour imaging scheme was then applied to study the subcortical cytoskeleton organization at synaptic sites. While a ~190 nm actin/betaII spectrin periodic lattice was recently reported lattice was recently reported along the axon and a subset of dendrites without spines^9^,^10^,^11^,^12^, its presence along dendrites decorated with spines and at synaptic sites is still uncharacterized. Our three-colour multilevelSTED nanoscopy of mature hippocampal neuronal cultures reveals that the periodic lattice is present in dendrites with spines, but absent at pre- and postsynaptic sites.

## Results

### STED Nanoscope

**Figure 1:**
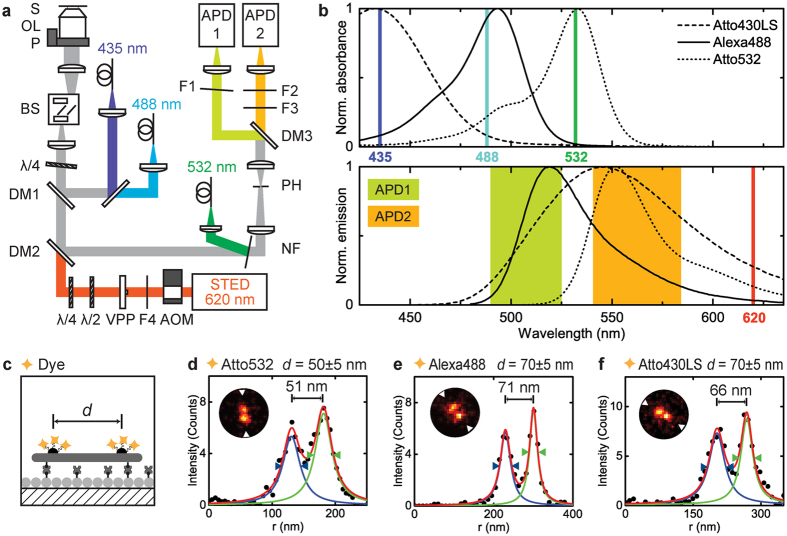
Setup and DNA origami imaging. (**a**) Schematic drawing of the main optical parts of the STED microscope: *S* Sample, *OL* objective lens, *P z*-piezo translator, *BS* beam scanner, *λ/2* half-wave plate, *λ/4* quarter-wave plate, *DM* dichroic mirror (DM1: 460DCXRU, DM2: ZT594RDC, DM3: T525LPXR), *NF* notch filter (532 nm Notch), *PH* pinhole, *VPP* vortex phase plate, *AOM* acousto-optical modulator, *F* filter (F1: 514/30, F2: 562/40, F3: 532 nm Notch, F4: 620/14), *APD* avalanche photodiode detector. (**b**) Normalized absorption and emission spectra of the dyes Atto430LS, AlexaFluor488 and Atto532 utilized for three-colour imaging, as well as related laser lines and detection windows. (**c**) Schematic drawing of a DNA origami molecule immobilized on a glass surface through biotin-neutravidin links. Two positions on the origami located at a distance *d* from each other are labelled with dye molecules. (**d**) Inset shows STED image of a single DNA origami molecule with spots marked by Atto532 molecules at *d* = 50 ± 5 nm. The line profile drawn across the DNA origami shown in the inset was fitted by two Lorentzian functions. Fitting yielded a spot separation distance of 51 nm with single spot full width at half maxima (FWHM) of <35 nm. (**e**) Same as (**d**), but for an origami with *d* = 70 ± 5 nm labelled with AlexaFluor488. The corresponding fit yielded a separation distance of 71 nm and single-peak FWHM of <50 nm. (**f**) Same as (**e**) for an Atto430LS-labelled origami. The corresponding fit yielded a separation distance of 66 nm and peak FWHM of <40 nm.

In the present implementation of multicolour STED nanoscopy, a single STED beam of 620 nm light with a doughnut-shape in the focal region is co-aligned to three Gaussian focal excitation spots of 435 nm, 488 nm and 532 nm (Fig. 1a and Methods Section). These excitation wavelengths cover the whole range of potentially interesting markers, notably dyes with an emission peak in the range of 520–560 nm including long-Stokes-shift dyes (Fig. 1b). Importantly, this broad distribution of emission spectra and hence of cross-sections for stimulated emission requires the adjustment of the STED power to achieve the same resolution with the same STED wavelength. To address this, we developed “multilevelSTED”, an approach that applies two (or more) different power levels during the acquisition of an image. MultilevelSTED provides a higher flexibility in the choice of parameters when optimizing the resolution and brightness to match the specific requirements of a particular multicolour specimen.

The fluorescence within the STED doughnut centre is collected by the objective lens used for excitation, and imaged onto a single confocal pinhole. From there, the signal is split into two spectral windows of 510 ± 15 nm (APD1) and 560 ± 20 nm (APD2), focused each on a separate avalanche photodiode (APD) (Fig. 1a). The combination of a single detection pinhole and a single STED doughnut ensures intrinsic co-alignment of the colour channels. Small misalignments of the excitation foci can be tolerated^8^. Fast scanning in the lateral directions is realized with a beam scanner consisting of four galvanometric scan mirrors^4^,^13^.

### STED at 620 nm with ~35 nm resolution

First, we tested a series of synthetic and protein-based fluorescent markers in 620 nm STED. The resolution performance for synthetic dyes was evaluated on DNA origami structures^14^ labelled at two positions with defined separation *d* (Fig. 1c). Spots on DNA origamis highlighted with Atto532 at *d* = 50 ± 5 nm could be clearly separated and showed individual full width at half maxima (FWHM) of <35 nm (Fig. 1d). Similarly, AlexaFluor488 and Atto430LS allowed the separation of two 70 ± 5 nm separated sites with single peak widths of <50 nm and <40 nm, respectively (Fig. 1e,f). Concerning protein-based markers for live cell imaging, STED at 620 nm imaged structures at resolutions of ~65 nm for EGFP, and ~55 nm for both YFP and Citrine (Supplementary Fig. 1).

Promising alternatives to fluorescent proteins for imaging in living samples are membrane-permeating synthetic dyes like silicon rhodamines^15^ in combination with bio-orthogonal labelling based on SNAP^16^-, CLIP^17^- and Halo^18^ -tag fusion proteins. However, up to now, to the best of our knowledge, there are no reports on fluorescent dyes with emission maxima around 560 nm suitable for imaging of structures within living cells. Therefore, we tested a water-soluble *6*′*-carboxy-Q*-rhodamine^19^ (*540R*) absorbing and emitting at 540 nm and 561 nm, respectively. This dye possesses a compact, zwitterionic structure with a relatively short distance between positive and negative charges, which is characteristic of cell-penetrating synthetic probes^20^. After incubation with its Halo-tag amine conjugate (*540R*-Halo, Supplementary Appendix 1), living HeLa cells expressing Halo-tag fusion proteins showed bright staining with low background and permitted 620 nm STED imaging with a resolution of ~45 nm (Supplementary Fig. 1d).

### Multicolour imaging with 620 nm STED

**Figure 2:**
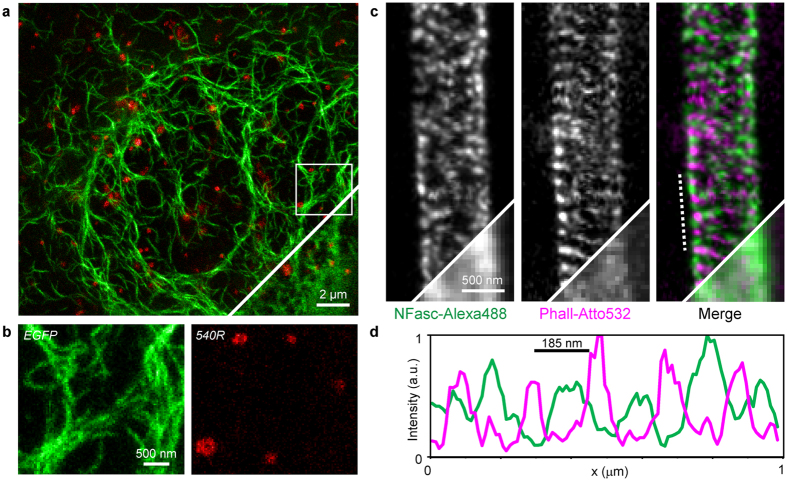
Dual-colour STED nanoscopy. (**a**) STED image of a living HeLa cell expressing vimentin-EGFP (green) and Pex3-Halo-tag stained with *540R*-Halo (red). The confocal counterpart is shown in the lower-right corner. (**b**) Close-ups of the region marked in (**a**) of the two colour channels, demonstrating the absence of cross-talk and the high signal-to-background ratio in both channels. (**c**) STED image of a fixed hippocampal axon stained against neurofascin with AlexaFluor488 (left) and actin with phalloidin-Atto532 (centre) reveals the characteristic periodic lattice. Corresponding confocal images are shown in the lower-right corner. (**d**) Line profiles drawn parallel to the line shown in (**c**) for the two channels illustrate the interplay of neurofascin and actin. Counts of three pixels perpendicular to the line were summed up. Data of all figure panels were smoothed with a 1.0 pixel wide Gaussian. No linear unmixing was performed.

After choosing a set of fluorescent markers, we explored their possible combinations for multicolour nanoscopy. To this end, we imaged living HeLa cells that expressed vimentin-EGFP and Pex3-Halo-tag (peroxisomal marker) fusion proteins, after staining them with *540R*-Halo (Fig. 2a,b). We applied the excitation lines 532 nm for reading out *540R* and 488 nm for reading EGFP (quasi) simultaneously along the fast scan direction, i.e., each image row was subsequently scanned multiple times with only one of the excitation beams being active during each line scan (line interleaved acquisition) (Supplementary Fig. 2a,b). Implementing multilevelSTED, 5 mW (0.13 nJ/pulse) of STED light were applied together with the 532 nm excitation beam (1.0 μW, 5 line repetitions) and 36 mW (0.90 nJ/pulse) together with 488 nm excitation (1.7 μW, 3 line repetitions), respectively. Ensuring that each fluorophore is treated with its optimal STED beam power improved resolution and brightness in both colour channels (Supplementary Fig. 3). By the same token, the overall STED light dose was reduced by 54% compared to standard STED recording modalities with a single power (set at 36 mW) for both channels. In combination with the two spectrally resolved detection channels (APD1 detecting EGFP and APD2 detecting *540R*), both structures could be super-resolved with a cross-talk below 1% in the raw data (Supplementary Fig. 2c).

For the imaging of fixed samples, the combination of AlexaFluor488 and Atto532 was evaluated (Fig. 1b). To this end, we labelled neurofascin with AlexaFluor488 and actin by phalloidin-Atto532 in cultured hippocampal neurons. Dual-colour STED imaging was carried out according to the same scheme as for the living samples. We could clearly discern the subtle ~190 nm actin pattern along axon initial segments^9^ and its local association with neurofascin^11^ (Fig. 2c,d).

Next, we evaluated the use of long-Stokes-shift dyes, and in particular Atto430LS, in combination with the dye pair AlexaFluor488-Atto532 for performing three-colour STED nanoscopy (Fig. 1b). Excitation wavelengths were again applied line by line, and the STED powers required for optimized imaging of AlexaFluor488 and Atto430LS in our samples was found to be in the same range (Supplementary Fig. 4a). Generally speaking, the implemented multilevelSTED approach allowed parameter optimization with high flexibility with respect to timing and the powers applied. The fluorescence emission was collected in four detection periods (APD1 along with 435 nm excitation, APD2 with 532 nm, APD1 and APD2 simultaneously with 488 nm). Thereby, Atto532 and Atto430LS signals could be separated with cross-talks ≤4%; AlexaFluor488 on its own featured ≤37% cross-talk (raw data) (Supplementary Fig. 4b). By linear unmixing^21^,^22^ all cross-talks could be reduced to ≤7% (Supplementary Fig. 4c,d).

**Figure 3:**
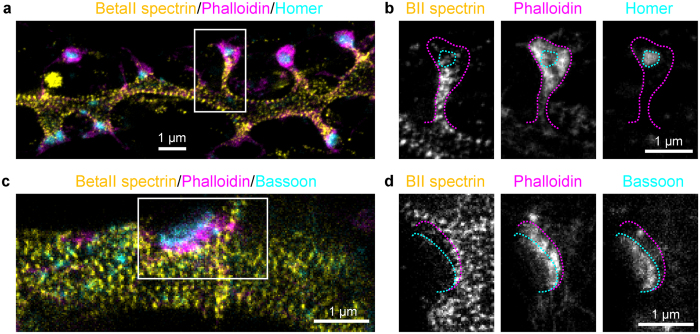
The subcortical periodic actin/betaII spectrin lattice is discontinued at synaptic sites. (**a**) Three-colour STED image of a dendrite decorated with spines and stained with betaII spectrin (AlexaFluor488, yellow), phalloidin (Atto532, magenta), and Homer (Atto430LS, cyan) shows the periodic spectrin organization. (**b**) Single-channel images of the spine indicated in (**a**). Magenta and cyan dashed lines highlight the shape of the spine and the position of the PSD, respectively. BetaII spectrin enters into the spine neck but does not reach the PSD. (**c**) Same as (**a**), but for the presynaptic site, identified by Bassoon staining instead of Homer. (**d**) Single channel images of the boxed area in (**c**). Magenta and cyan dashed lines highlight the position of the actin cage and of Bassoon, respectively. All panels of the figure show linear unmixed STED data, smoothed with a 1.0 pixel wide Gaussian function. Raw data of the image in panel (**a**) is shown in Supplementary Fig. 4d.

Next, we applied our multicolour STED approach for imaging the cytoskeletal organization in mature hippocampal neurons. Concretely, we targeted the subcortical proteins betaII spectrin (stained by AlexaFluor488) and actin (phalloidin-Atto532)^9^,^12^, together with the pre-synaptic marker Bassoon and its post-synaptic counterpart Homer (Atto430LS). All three colour channels were bright and well resolved, with the respective structures identified unequivocally (Fig. 3). Therefore, the proposed and demonstrated instrument proved suitable for further biological studies.

### Cytoskeletal organization at synaptic sites of hippocampal neurons

In recent years a ~190 nm actin/betaII spectrin periodic subcortical lattice was discovered along the distal axons^9^ and the dendrites^11^,^12^ of hippocampal neurons by STORM and STED nanoscopy. Indeed, the periodic subcortical lattice was recently shown to be a ubiquitous feature of neurites in both the central and peripheral nervous systems^23^. However, many questions are still open, and in particular: (i) is the periodic lattice still present in dendrites that have lost their regular tubular shape due to the outgrowth of spines? And (ii) is the lattice present also at pre- and post-synaptic sites? We took advantage of the capability to perform three-colour recordings to answer these questions. Actin is highly enriched in the dendrites and especially in the spine heads^24^ (Fig. 3a), and therefore the identification of the fine subcortical structure with phalloidin staining was difficult. Nevertheless, betaII spectrin shows a sharp periodic organization along all the dendrites decorated with spines, indicating the presence of the subcortical actin/spectrin lattice also in mature neurons. Phalloidin staining intercalating the spectrin pattern was observed only infrequently, due to the presence of numerous actin bundles running along the dendrites, shading the fine subcortical arrangement (Supplementary Fig. 5a,b). The betaII spectrin lattice enters into the thicker spine necks, but is generally absent from postsynaptic densities (PSD) as identified by Homer staining (Fig. 3a,b and Supplementary Fig. 5c). At times, betaII spectrin puncta are observed next to the Homer hotspots, where lower actin levels are observed concomitantly. Similarly, also at presynaptic sites, identified by Bassoon staining, betaII spectrin content appears reduced and the periodic pattern is discontinued (Fig. 3c,d and Supplementary Fig. 5d). In most of the cases, an actin enrichment is observed adjacent to the active zone but not at Bassoon hotspots. Therefore, even if the actin/betaII spectrin subcortical periodic lattice is present in all neurites of mature neurons, it is absent from pre- and postsynaptic sites.

## Discussion and Conclusions

The absence of the actin/betaII spectrin lattice at synaptic sites can be explained with the need for plasticity and rapid re-arrangements occurring at these highly specialized locations. Morphological and functional changes would indeed require the disassembly of the subcortical lattice^25^. Furthermore, the presence of a tight lattice might impede the fusion of vesicles with the membrane^26^. On the other hand, we cannot rule out that other spectrin isoforms are specifically enriched at synaptic sites and substitute for the scaffolding function of betaII spectrin^27^,^28^, similarly to what is found in the axon initial segment and at the nodes of Ranvier with betaIV spectrin^12^,^29^.

Regarding the potential of the described STED implementation, it was demonstrated to perform well for different markers and imaging challenges. STED at 620 nm wavelength is viable with several synthetic and protein-based markers, whereby the obtained resolution of down to ~35 nm with synthetic dyes is similar to those provided by STED systems of 590 nm wavelength. Importantly, in combination with EGFP and the cell-penetrating Q-rhodamine dye *540R*, the 620 nm line offers a straightforward way of performing live-cell two-colour nanoscopy with high channel discrimination. With ongoing dye development, three-colour imaging with live-cell compatible synthetic markers should also be possible in the not too distant future.

For co-aligned three-colour nanoscopy of fixed cells, we combined two “standard” dyes with a long-Stokes-shift dye. The potential of this approach has been demonstrated by imaging of hippocampal neurons. In fact, this strategy can also be followed for commercially available ~775 nm STED systems with three excitation wavelengths around 490 nm, 560 nm and 640 nm. Corresponding dyes include Atto490LS, STAR580/AlexaFluor594 and STAR635 (Supplementary Fig. 6). The low cross-talk of our imaging scheme can be further reduced by a linear unmixing routine that may be even directly implemented in the microscope software. Therefore, imaging of two standard dyes in combination with a long-Stokes shift dye forms a basis for establishing routine applications without the need for elaborate setup alignments, extended data processing, or equipment for fluorescence lifetime separation^3^. Moreover, with multilevelSTED, we have presented a strategy that both optimizes the image quality and reduces the required STED light dose, which is especially beneficial when performing live-cell nanoscopy and, in general, for reducing the photobleaching. Here, the STED power was modulated along the fast scan axis. However, in appropriately interleaved excitation schemes the power can also be varied pixel- or even pulse-wise using acousto- or electro-optical modulators. In future implementations, the number of selectable power levels could be equal to or even exceed the number of excitation lines.

Our imaging scheme can also be implemented in a 3D STED nanoscopy scheme. Since the gain in resolution is effected with a single STED wavelength for all colour channels, a second STED beam engineered as ‘*z*-doughnut’^30^ (bottle beam) is readily added or, even better, the corresponding phase shift can be imprinted on the same STED beam. Resolution enhancement along all three dimensions would be helpful for a more detailed characterization of the complex 3D structure of synaptic sites. Furthermore, the presented setup allows the implementation of continuous-wave beams needed for reversible saturable optical fluorescence transition (RESOLFT)^31^ and Protected STED^32^ nanoscopy with reversibly switchable EGFP variants^33^.

In conclusion, we demonstrated that a compact 620 nm STED laser provides superresolution down to 35 nm as well as versatility for dual- and three-colour recordings of living and fixed cells, respectively. Furthermore, we found that adapting the STED beam intensities to each individual colour channel (multilevelSTED) improves multicolour recording and reduces the overall STED light dose. Last but not least, we showed the usefulness of our STED nanoscope by revealing the absence of the actin/betaII spectrin subcortical lattice at synaptic sites of hippocampal neurons.

## Materials and Methods

### STED nanoscope

STED pulses (620 nm, ~600 ps FWHM, 40 MHz) were delivered by a fibre laser (MPB Communications Inc., Montreal, Quebec, Canada). A part of the experiments were carried out with a Raman-shifted fibre laser (Rainbow prototype, IPG Photonics, Mountain View, CA, USA) operating at 618 nm with a repetition rate of 20 MHz (pulse width similar to the MPB laser). Both STED lasers provided similar results. Pulsed diode lasers (488 nm: PicoTA, Toptica Photonics, Graefelfing, Germany and PicoQuant, Berlin, Germany; 435 nm: LDH-D-C-430; 532 nm: LDH-P-FA-530, both PicoQuant) served as excitation light sources and were triggered by the STED laser. All lasers were spectrally filtered, passed through acousto-optical modulators (AOMs) and were subsequently coupled into polarization-maintaining single-mode fibres (exception: the MPB laser was not fibre-coupled). Fast alternation between two levels of the analogue voltage at the AOM defining the STED power was accomplished by home-built electronics. STED light was passed through a vortex phase-plate (VPP1a, RPC Photonics, Rochester, NY, USA, 620 nm vortex mask) giving rise to a doughnut-shaped focal spot. Beams were combined by dichroic mirrors and notch filters and coupled into the back aperture of an oil-immersion objective lens with NA 1.4 (HCX-PL-APO 100x/1.4-0.7 OIL CS, Leica Microsystems, Wetzlar, Germany). Scanning in the lateral directions was accomplished by a ‘Quad-Scanner’^13^, which consists of four galvanometric scan mirrors. For fine focus control and scanning in the axial direction, the objective lens was moved by a piezo translator (z-piezo, Mipos 100PL CAP, Piezosystem Jena, Jena, Germany). The fluorescence light was collected by the same objective lens, de-scanned and separated from the laser light, and focussed onto a pinhole of variable size (MPH16, Thorlabs, Newton, NJ, USA). Depending on the sample, the pinhole diameter was varied between sizes corresponding to 0.7–1.0 times the Airy disk at this position. The transmitted fluorescence light was then further spectrally separated, filtered and focussed on two avalanche photodiodes (each SPCM-AQRH-13, Excelitas, Waltham, MA, USA). The detection was time-gated on the μs- and ns-timescale by home-built electronics and by an FPGA card (PCIe-7852R, National Instruments, Austin, TX, USA), respectively. Image acquisition and microscope control were performed with the software ImSpector (Max-Planck Innovation).

### Image acquisition and analysis

All power values stated in this work refer to the power entering the back aperture of the objective lens, meaning that the actually applied power is typically 10–30% lower due to losses at the optical interfaces presented by the objective lens and the sample. Linear unmixing was performed with the SpectralUnmixing plugin (rsbweb.nih.gov/ij/plugins/spectral-unmixing.html) of ImageJ (imagej.nih.gov/ij/) and was applied where indicated. The unmixing matrix was obtained from measurements with the same parameters on single-colour stainings. No deconvolution was performed. Cross-talks of colour channels were determined by calculating the average pixel counts normalized to the brightest channel. Where indicated, images were smoothed by convolution with a 1.0 pixel wide Gaussian using ImSpector. Some neuron images were rotated using bicubic interpolation in ImageJ. Line profiles were fit using the multi-peak fitting function in OriginPro2015. Pixel counts of repetitive scans along the fast scan axis were summed up and cumulative counts are reported.

The imaging parameters for the data presented were as follows:

- Figure 1: Customized DNA origami structures were obtained from GattaQuant, Braunschweig, Germany. (d) 10 scans along fast scan axis with 532 nm excitation (0.9 μW) + 620 nm STED (29 mW), detection with APD2. Pixel dwell time: 4.5 μs. Pixel size: 10 nm. (e) 8 scans along fast scan axis with 488 nm excitation (1.7 μW) + 620 nm STED (39 mW), detection with APD1 and APD2, and signals were summed. Pixel dwell time: 2 μs. Pixel size: 16.5 nm. (f) 3 scans along fast scan axis with 435 nm excitation (0.9 μW) + 620 nm STED (40 mW), detection with APD1 and APD2, and signals were summed. Pixel dwell time: 20 μs. Pixel size: 15 nm. Images smoothed by convolution with a 1.0 pixel wide Gaussian. Line profiles taken on raw image data, averaged over 2 or 3 pixels perpendicular to the direction of the profile.
- Figure 2: (a) Sequence along the fast scan axis: 3 scans with 488 nm excitation (1.7 μW) + 620 nm STED (36 mW), detection with APD1; 5 scans with 532 nm excitation (1.0 μW) + 620 nm STED (5 mW), detection with APD2. Pixel dwell time: 13 μs + 5 μs break. Frequency of repetitions along fast scan axis: 50 Hz. Progression along slow scan axis: 6.2 Hz. Pixel size: 30 nm. (**c**) Sequence along fast scan axis: 3 scans with 488 nm excitation (1.8 μW) + 620 nm STED (36 mW), detection with APD1; 3 scans with 532 nm excitation (1.0 μW) + 620 nm STED (15 mW), detection with APD2. Pixel dwell time: 7 μs + 5 μs break. Pixel size: 20 nm. Images were smoothed with 1.0 pixel wide Gaussian.
- Figure 3: (a,c) Sequence along fast scan axis: 3 scans with 532 nm excitation (1.7 μW) + 620 nm STED (15 mW), detection with APD2; 3 scans with 488 nm excitation (2.4 μW) + 620 nm STED (42 mW), detection with APD1 and APD2; 4 scans with 435 nm excitation (3.0 μW) + 620 nm STED (42 mW), detection with APD1. Pixel dwell time: 7 μs + 5 μs break. Pixel size: 25 nm. Images were linearly unmixed (see above) and smoothed with 1.0 pixel wide Gaussian.

### Cell Culture, transfection and labelling of living cells

HeLa cells were plated on glass coverslips and transfected on the next day with a plasmid encoding a fusion of a fluorescent protein or Halo-tag with the protein of interest. About 24 h after transfection, cells were washed and imaged in HEPES-buffered Dulbecco’s modified Eagle’s medium (HDMEM) without phenol red. Cells transfected with a Halo-tag-containing plasmid were incubated in a 1 μM solution of *540R*-Halo in DMEM for 20–30 min under growth conditions. After that, cells were washed in HDMEM and incubated for another 20–30 min in DMEM under growth conditions. Cells were imaged in HDMEM without phenol red.

### Primary hippocampal neuron culture preparation

Cultures of hippocampal neurons were prepared from Wistar rats of mixed sex at postnatal day P0–P1 in accordance with Animal Welfare Law of the Federal Republic of Germany (Tierschutzgesetz der Bundesrepublik Deutschland, TierSchG) and the Regulation about animals used in experiments (1^st^ August 2013, Tierschutzversuchsverordnung). For the procedure of sacrificing rodents for subsequent preparation of any tissue, all regulations given in §4 TierSchG are followed. Since sacrificing of animals is not an experiment on animals according to §7 Abs. 2 Satz 3 TierSchG, no specific authorization or notification is required. Cells were plated on coverslips coated with 100 μg/mL polyornithine (Sigma-Aldrich, cat. P3655) and 1 μg/mL laminin (BD Bioscience, cat. 354232). Neuronal cultures were maintained in Neurobasal medium (Gibco, cat. 21103049) supplemented with 2% B27 serum-free supplement (Gibco, cat. 17504044), 2 mM L-glutamine (Gibco, cat. 25030) and pen/strep (100 units/mL and 100 μg/mL, respectively, BiochromAG, cat. A2213). On the day after plating, 5 μM cytosine β-D-arabinofuranoside (Sigma, cat. C1768) was added to the cultures. For this study, neuronal cultures at 17–30 days *in vitro* in which spines were fully developed were used.

### Immunostaining

Cells were washed with PBS and fixed in 4% PFA in PBS (pH 7.4) for 20 min at room temperature (RT), quenched with NH_4_Cl and glycine (100 mM each) for 5 min, permeabilized with 0.1% Triton X-100 for another 5 min, and blocked with BSA 1% in PBS for 30 min. Both primary and secondary antibodies and phalloidin incubations were performed in PBS for 1 h at RT or overnight at 4 °C. Samples were mounted in Mowiol supplemented with DABCO.

The antibodies used in this study are: anti-pan-neurofascin (UC Davis/NIH NeuroMab Facility, clone A12/18 cat. 75–172, 1:400 dilution); anti-betaII spectrin (BD Biosciences, cat. 612563, 1:400 dilution); anti-Homer 1 (Synaptic Systems, cat. 160 003, 1:200 dilution); anti-Bassoon (Synaptic Systems, cat. 141 003 and 141 004, 1:200 dilution); anti-mouse AlexaFluor488 (Invitrogen, cat. A11001, 1:200 dilution); anti-mouse STAR635 (Abberior, cat. 2-0002-002-0, 1:100 dilution). Anti-rabbit secondary antibody (Dianova, cat. 111-005-003) and anti-guinea-pig secondary antibody (Dianova, cat. 706-005-148) were custom-labelled with Atto430LS dye (AttoTech, cat. AD 430LS-31) or Atto490LS dye (AttoTech, cat. AD 490LS-31). Phalloidin was coupled to Atto532 (AttoTech, cat. AD 532-81, 1:100 dilution) or to STAR580 (Abberior, cat. 2-0205-005-6, 1:100 dilution).

## Additional Information

**How to cite this article**: Sidenstein, S. C. *et al.* Multicolour Multilevel STED nanoscopy of Actin/Spectrin Organization at Synapses. *Sci. Rep.* **6**, 26725; doi: 10.1038/srep26725 (2016).

## Supplementary Material

Supplementary Information
