# Supplementary material for: Multicolour Multilevel STED nanoscopy of Actin/Spectrin Organization at Synapses
Source: Sci Rep. 2016 May 25;6:26725. doi: 10.1038/srep26725 (PMC4879624; doi:10.1038/srep26725)
Supplement: Supplementary Information [file srep26725-s1.pdf]

Supporting Information

Multicolour Multilevel STED nanoscopy of Actin/Spectrin Organization at Synapses

Sven C. Sidenstein[1], Elisa D'Este[1], Marvin J. Böhm[1,2], Johann G. Danzl[1], Vladimir N. Belov[1] & Stefan W. Hell[1]*

[1]Max Planck Institute for Biophysical Chemistry, Department of NanoBiophotonics, Am Fassberg 11, 37077 Göttingen, Germany

[2]Present address: Georg-August-Universität Göttingen, Institut für Organische und Biomolekulare Chemie, Tammannstraße 2, 37077 Göttingen, Germany

*Correspondence to shell@mpibpc.mpg.de

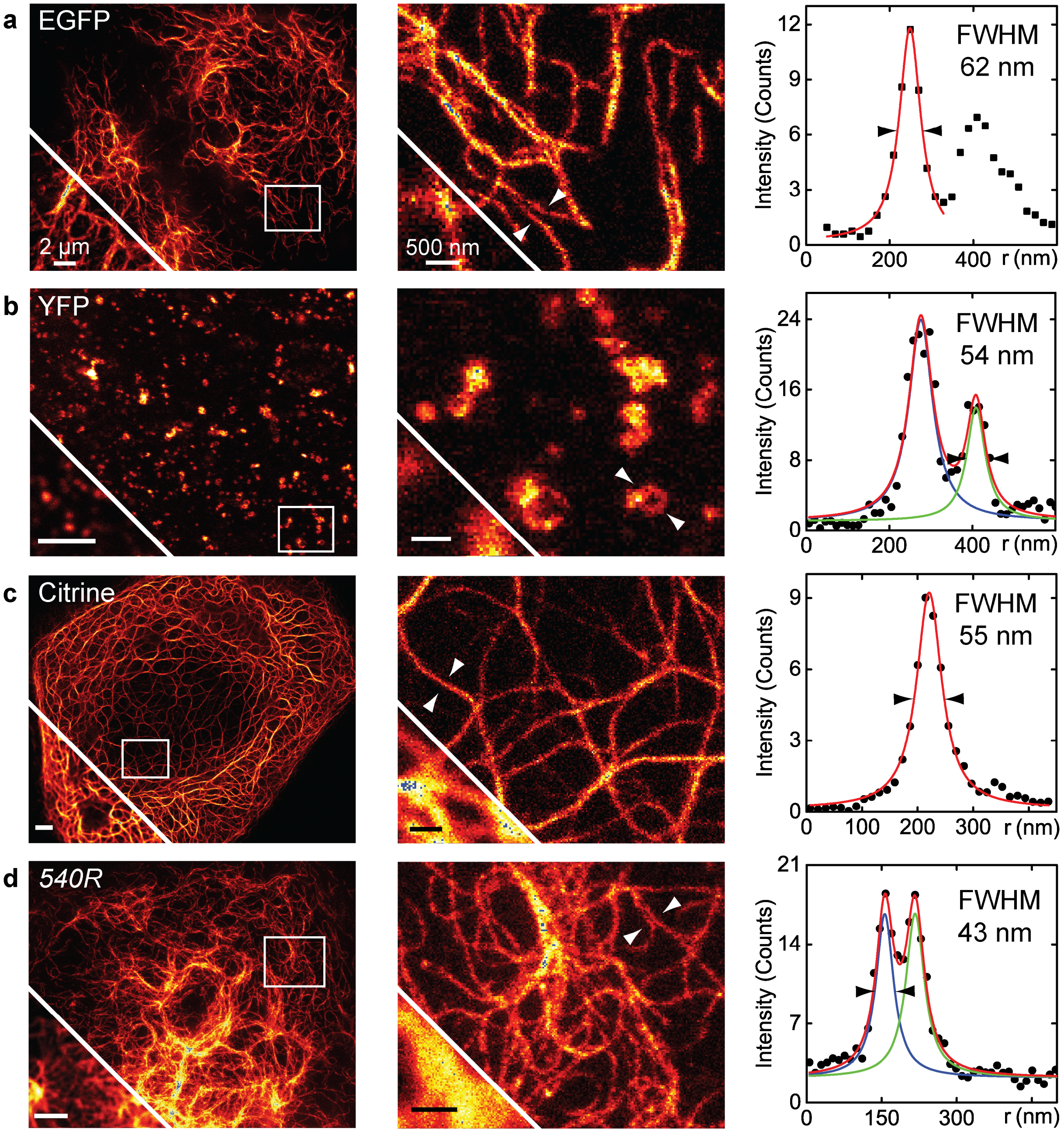

Supplementary Figure 1: Fluorescent proteins and *540R* dye in 620 nm STED nanoscopy. Raw STED images, along with confocal sections in lower-left corner, of living HeLa cells expressing (**a**) vimentin-EGFP, (**b**) Caveolin1-YFP, (**c**) Keratin-Citrine, and (**d**) vimentin-Halo stained with *540R*, respectively. Images in the middle column show close-ups of the region marked in the respective left image. The line profiles (right column) of filaments indicated by arrowheads in the zoomed views were fitted to Lorentzian functions depicted in the respective diagrams. The

obtained full width at half maximum (FWHM) values give an approximation of the optical resolution. Features at this level of resolution and contrast can be found in multiple locations within the imaged field. Scale bars left: 2 µm; scale bars right: 500 nm.

Imaging parameters: (**a**) 3 scans along fast scan axis with 488 nm excitation (1.7 µW) + 620 nm STED (36 mW, 40 MHz, 0.90 nJ/pulse), detection with APD1. Pixel dwell time: 13 µs + 5 µs break. Pixel size: 30 nm. (**b**) 2 scans along fast scan axis with 488 nm excitation (1.1 µW, 20 MHz) + 618 nm STED (16 mW, 20 MHz, 0.80 nJ/pulse), detection with one APD filtered with a 550/49 bandpass. Pixel dwell time: 30 µs. Pixel size: 20 nm. (**c**) 2 scans along fast scan axis with 488 nm excitation (1.7 µW) + 620 nm STED (26 mW, 40 MHz, 0.65 nJ/pulse), detection with APD1 and APD2 and signals were summed up. Pixel dwell time: 10 µs + 5 µs break. Pixel size: 23 nm. (**d**) 2 scans along fast scan axis with 532 nm excitation (2.6 µW, 20 MHz) + 618 nm STED (10 mW, 20 MHz, 0.50 nJ/pulse), detection with APD2. Pixel dwell time: 25 µs. Pixel size: 18 nm. Line profiles were averaged over **a** 10 pixels, **b** 2 pixels, **c** 10 pixels, **d** 6 pixels perpendicular to the direction of the profile.

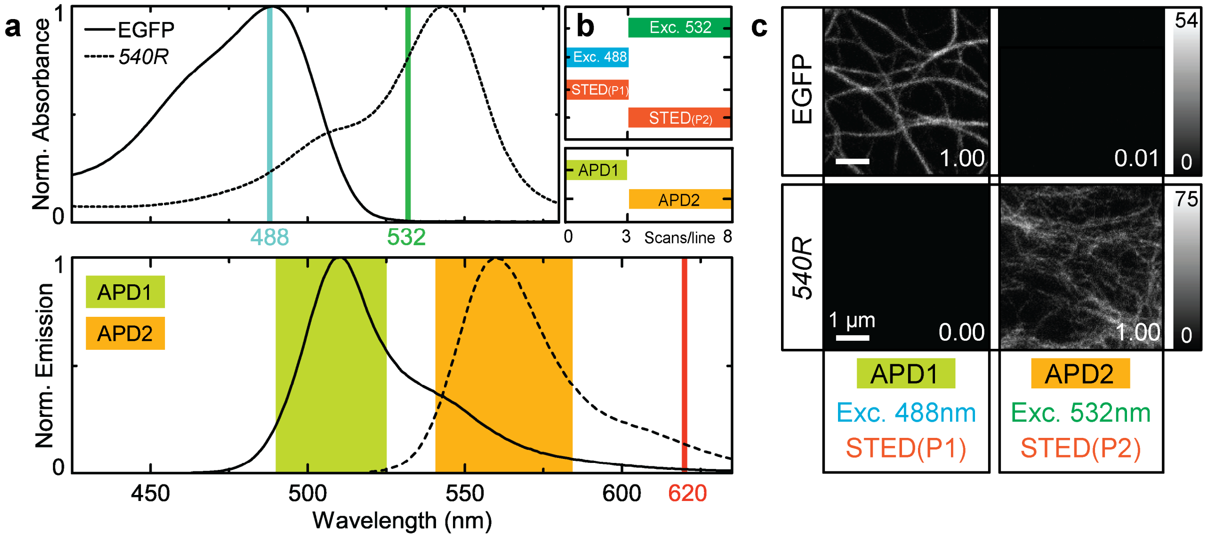

Supplementary Figure 2: Live dual-colour STED nanoscopy scheme. (**a**) Normalized absorbance and emission spectra of the fluorescent protein EGFP and the membrane-permeating dye *540R*-Halo along with the applied laser lines and detection windows of APD1 (green area) and APD2 (orange area). (**b**) Sequence of line steps (i.e. scan repetitions along the fast scanning direction) carried out for imaging of EGFP and *540R*. Pixel counts of line repetitions were summed. P1 and P2 denote different STED powers. (**c**) Application of the imaging sequence described in **b** on single colour stainings of living HeLa cells expressing keratin-EGFP (upper row) or vimentin-Halo-tag stained with *540R* (lower row). Images are raw STED data with the same colourmap settings for the two detection channels. Values stated in the images refer to average pixel counts normalized to the brighter channel indicating signal cross-talk of ≤1% for both markers.

Imaging parameters: (**c**) Sequence along fast scan axis: 3 scans with 488 nm excitation (1.7 µW) + 620 nm STED (P1: 36 mW), detection with APD1; 5 scans with 532 nm excitation (1.0 µW) + 620 nm STED (P2: 12 mW), detection with APD2. Pixel dwell time: 13 µs + 5 µs break. Pixel size: 30 nm. Figure shows raw data.

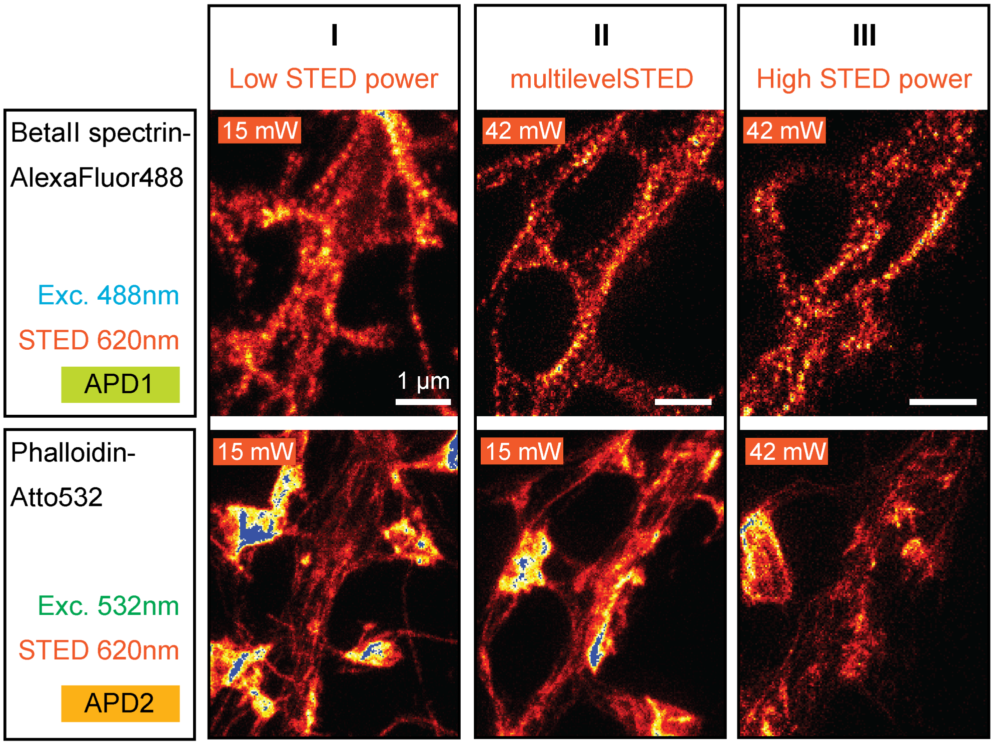

Supplementary Figure 3: Effect of multilevelSTED. Three different regions I-III along the same dendrite of a hippocampal neuron were imaged either with fixed STED power (I and III) or with multilevelSTED (II). For each region, the two stained structures betaII spectrin-AlexaFluor488 (upper row) and phalloidin-Atto532 (lower row) were recorded line-wise with the settings shown in the boxes on the left. In region I, a low STED power of 15 mW (0.38 nJ/pulse) was used for imaging both markers, whereby optimal resolution and brightness were only obtained for the Atto532 channel. The spectrin pattern in the AlexaFluor488 channel remained largely unresolved. In region III, a higher STED power of 42 mW (1.0 nJ/pulse) was applied, enabling the high-resolution rendition of the spectrin lattice. However, the fine actin filaments pervading the dendrite appeared too dim due to the overshot STED power applied to Atto532. Only in region II, where the respective optimal STED powers for both markers were applied using the multilevelSTED method, images were well-resolved and bright for both structures. Concomitantly, in region II STED dose was reduced by 26% compared to region III.

<u>Imaging parameters:</u> Scan sequence along fast scan axis: 3 scans with 532 nm excitation (1.7 µW) + 620 nm STED (15 mW or 42 mW), detection with APD2; 3 scans with 488 nm excitation (2.4 µW) + 620 nm STED (15 mW or 42 mW), detection with APD1. Pixel dwell time: 7 µs + 5 µs break. Pixel size: 25 nm. Figure shows raw data.

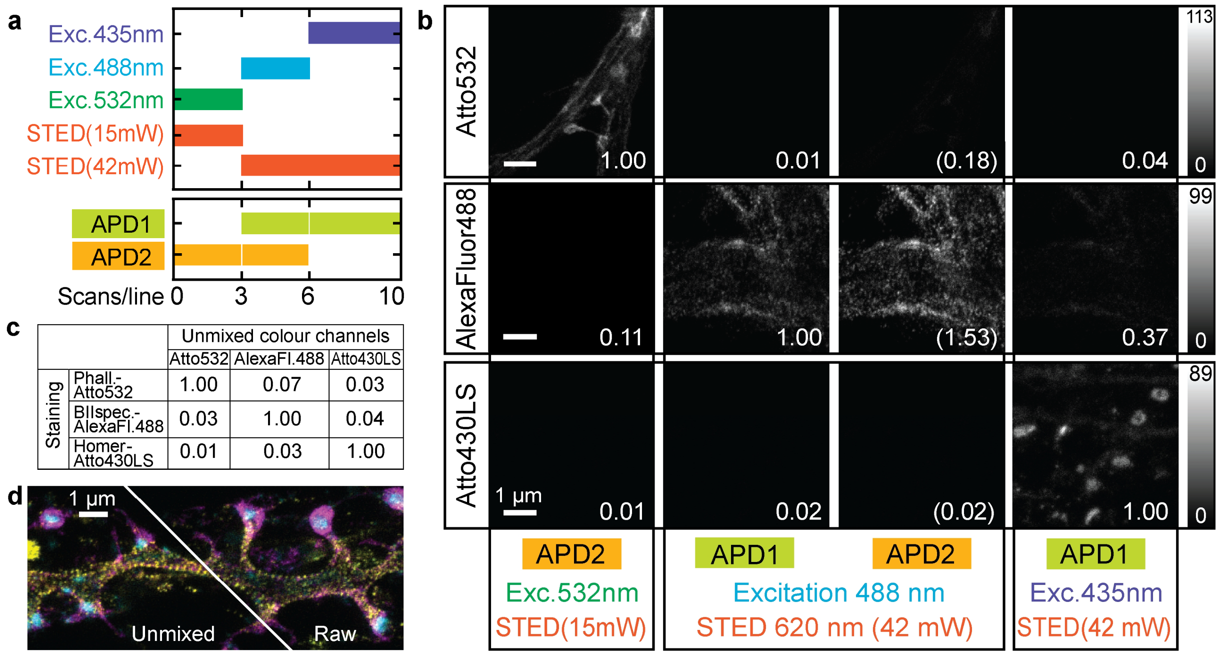

		Unmixed colour channels		
		Atto532	AlexaFl.488	Atto430LS
Staining	Phall.-Atto532	1.00	0.07	0.03
	BIIspec.-AlexaFl.488	0.03	1.00	0.04
	Homer-Atto430LS	0.01	0.03	1.00

Supplementary Figure 4: Three-colour STED nanoscopy scheme. (**a**) Sequence of repetitions along the fast scanning axis carried out for three-colour imaging. The pixel counts of lines with constant laser settings were accumulated. (**b**) Application of the imaging sequence shown in panel **a** on single colour stainings of hippocampal neurons: phalloidin-Atto532 (first row), betaII spectrin-AlexaFluor488 (second row) and Homer-Atto430LS (third row). The panel shows raw STED images together with average pixel counts normalized to the brightest channel. Colourmaps were adjusted to the brightest channel. Counts collected with APD2 during 488 nm excitation can be mainly attributed to AlexaFluor488 and can be used, as needed, for linear unmixing. (**c**) Matrix of normalized average pixel counts of the linearly unmixed datasets shown in **b**. For linear unmixing all four detection channels per measurement were used. Cross-talk could be reduced to ≤7% for all markers. (**d**) Raw STED data of the experiment shown in **Fig. 3a** (magenta: phalloidin-Atto532; yellow: spectrin-AlexaFluor488, cyan: Homer-Atto430LS). Linear unmixed data is depicted on the left demonstrating mainly an improved signal in the AlexaFluor488 channel.

Imaging parameters: (**b**) Sequence along fast scan axis: 3 scans with 532 nm excitation 1.7 µW) + 620 nm STED (15 mW), detection with APD2; 3 scans with 488 nm excitation (2.4 µW) + 620 nm STED (42 mW), detection with APD1 and APD2; 4 scans with 435 nm excitation (3.0 µW) + 620 nm STED (42 mW), detection with APD1. Pixel dwell time: 7 µs + 5 µs break. Pixel size: 25 nm. Panel shows raw data. (**d**) Same dataset shown in **Fig. 3a**.

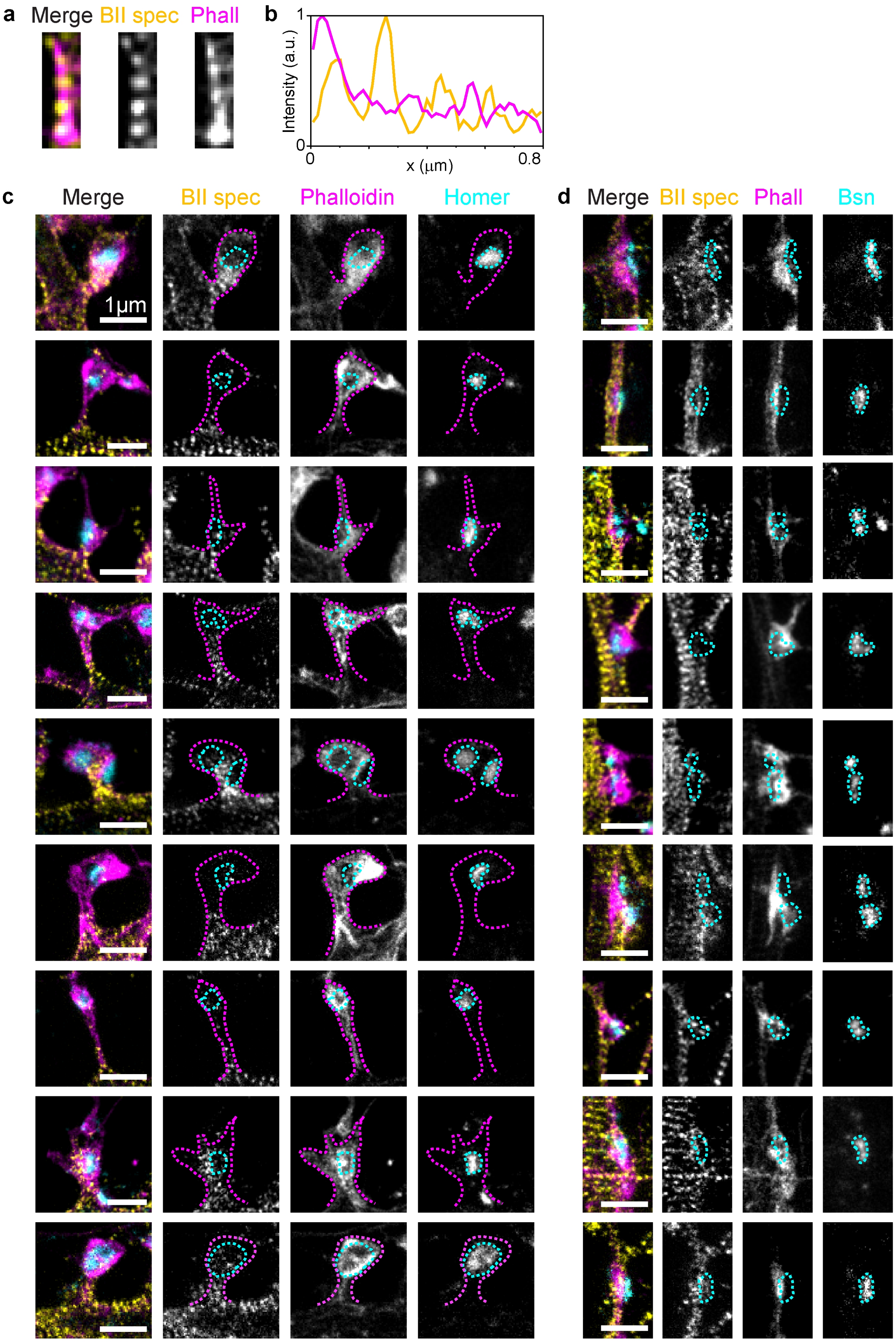
a
Merge
BII spec
Phall
b
Intensity (a.u.)
1
0
0
0.8
x (µm)
c
Merge
BII spec
Phalloidin
Homer
1µm
d
Merge
BII spec
Phall
Bsn

Supplementary Figure 5: Actin and betaII spectrin organization at pre- and postsynaptic sites. (**a**) Close-up of **Fig. 3a** showing the actin/betaII spectrin alternating pattern along mature dendrites in between the spines (300x900 nm^2 section). (**b**) Line profile along **a** showing the interspaced peaks. A line profile of five-pixel width was extracted from the raw data. (**c**) Gallery of dendritic spines stained against betaII spectrin (AlexaFluor488, yellow), phalloidin (Atto532, magenta), and Homer (Atto430LS, cyan). Merged and corresponding single channel images are shown. Magenta and cyan dashed lines highlight the shape of the spine and the position of the PSD, respectively. (**d**) Same as **c**, but for the presynaptic site, identified by Bassoon (Bsn) staining. The cyan dashed line highlights the position of the Bassoon puncta. The figure shows linear unmixed STED data, smoothed by convolution with a 1.0 pixel wide Gaussian.

Imaging parameters: Scan sequence along fast scanning axis: 3 scans with 532 nm excitation (1.7 µW) + 620 nm STED (15 mW), detection with APD2; 3 scans with 488 nm excitation (2.4 µW) + 620 nm STED (42 mW), detection with APD1 and APD2; 4 scans with 435 nm excitation (3.0 µW) + 620 nm STED (42 mW), detection with APD1. Pixel dwell time: 7 µs + 5 µs break. Pixel size: 25 nm or 30 nm.

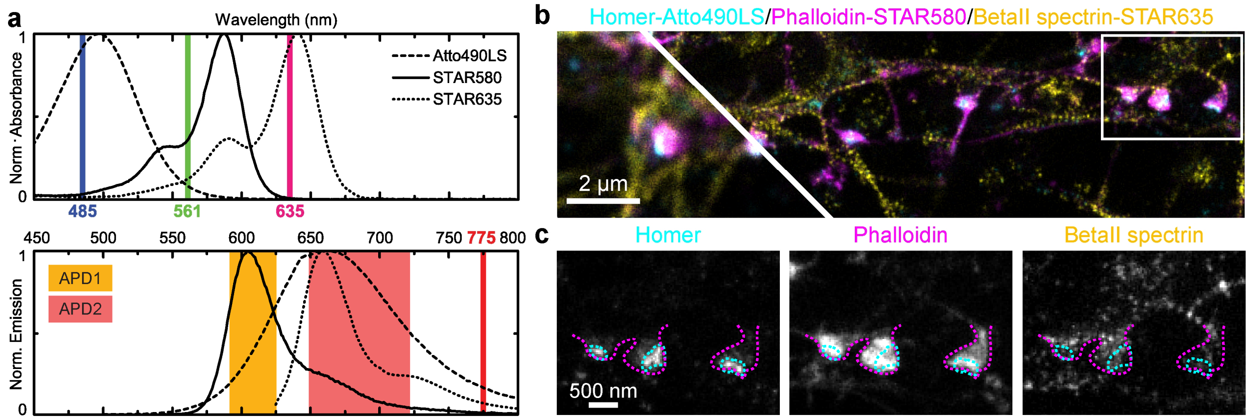

Supplementary Figure 6: Three-colour nanoscopy with a commercial 775 nm STED system. (**a**) Normalized absorption and emission spectra of the utilized dyes Atto490LS, STAR580, and STAR635 together with the applied excitation and STED wavelengths. Fluorescence was registered by two APDs (APD1: 605-625 nm; APD2: 650-720 nm). (**b**) Raw STED image of hippocampal neurons stained with Homer-Atto490LS, phalloidin-STAR580 and betaII spectrin-STAR635. A confocal comparison is depicted in the lower-left corner. (**c**) Single colour channels of the raw STED image of the region marked in **b**. Homer-Atto490LS (left, 485 nm excitation) and spectrin-STAR635 signals (right, 635 nm excitation) were detected with APD2. Phalloidin-STAR580 (centre, 561 nm excitation) was recorded with APD1. Excitation wavelengths were applied line-wise. The STED power, which effectively acts on the S1 state of dye molecules excited with the 635 nm beam, was reduced by shifting the rising edge of the STED pulse (width ~1 ns) several hundred ps ahead of the excitation pulse. As a consequence, the effect of multilevelSTED was partially simulated in this measurement.

<u>Imaging parameters:</u> (**b**) Measurements were performed with an unmodified, commercial STED system (2C STED775, Abberior Instruments, Göttingen, Germany) equipped with the excitation lines 485 nm, 561 nm and 640 nm, a STED laser at 775 nm (40 MHz) and two gated detection channels. Sequence along fast scan axis: 3 scans with 640 nm excitation + 775 nm STED (pulse timing described above), detection with APD2; 3 scans with 561 nm excitation + 775 nm STED, detection with APD1; 3 scans with 485 nm excitation + 775 nm STED, detection with APD2. Pixel dwell time: 7 µs. Pixel size: 30 nm.

Supplementary Appendix 1: Synthesis and isolation of 6´-Carboxy-Q-Rhodamine (*540R*) and its conjugate with Halo-tag amine. A mixture of 5'- and 6'-carboxy-Q-rhodamines (**3a,b** in **Scheme S1**) was synthesized by condensation of 7-hydroxy-1,2,3,4-tetrahydroquinoline (**1**) with trimellitic anhydride (**2**) in phosphoric acid[1]. Only 6'-carboxy isomers of the fluorophores were shown to provide successful labelling of proteins by means of HaloTag® and SNAP-tag® self-labelling techniques.[2,3] Therefore, we separated the mixture of 5'- and 6'-carboxy-Q-rhodamines, isolated the pure 6'-carboxy-isomer (***540R* = 3a**; for details, see reference[1]) and attached the Halo-tag amine to 6'-position of the pendant phenyl ring employing the bis(oxyethylene) linker **5** identified as optimal in the original report from Promega Corp. (**Scheme S1**)[4,5]. ***540R* = 3a:** UV-Vis spectrum (H_2O+10% PBS-buffer, pH 7.4): $\lambda_{max.}$ = 540 nm (absorption), ε = 70 300 M^{-1} cm^{-1}, λ_{max}= 561 nm (emission, QY = 0.97, reference - Rhodamine 6G with QY = 0.9). The amidation reaction was carried out according to the reported procedure[6,7]. Briefly, addition of *N*-hydroxysuccinimide (NHS), triethyl amine and HATU in small portions as solutions in DMF to the solution of **3a** (***540R***) in DMF was monitored by HPLC, until 64% of the starting material was converted into a mixture of mono *N*-hydroxy succimimidyl- (NHS) and di-NHS esters (and two unidentified side products). Addition of Halo-tag amine **5** led to the formation of the required amide **4** (**Scheme S1**). Compound **4** was isolated by HPTLC ($CHCl_3$:MeOH:H_2O = 70:27:3, R_f = 0.44-0.52 depending on the air humidity).

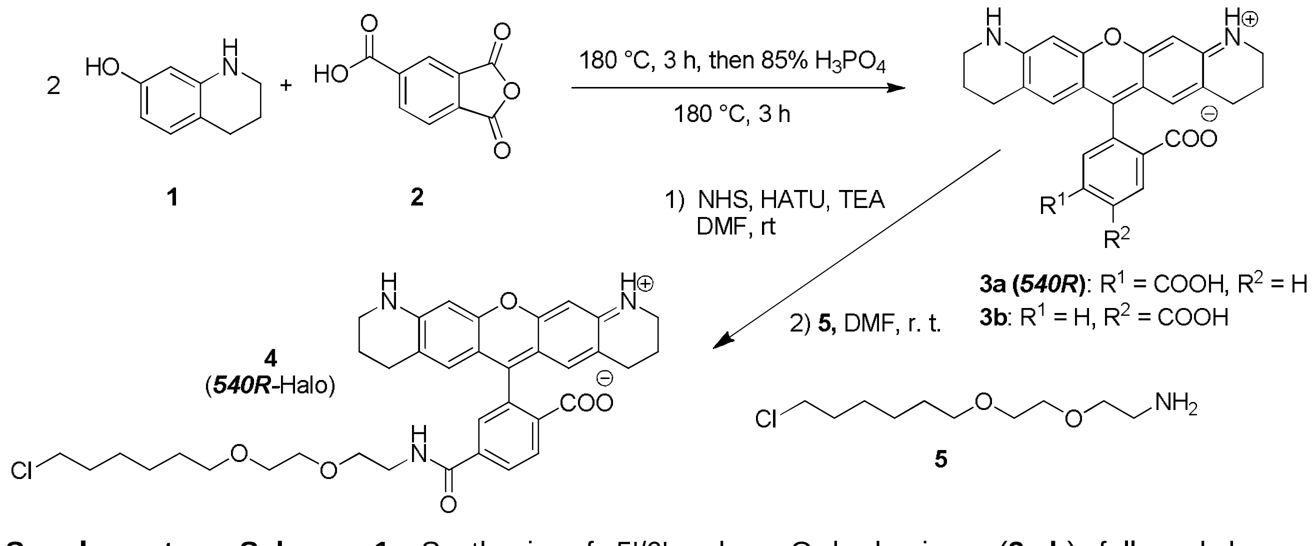

Supplementary Scheme 1. Synthesis of 5'/6'-carboxy-Q-rhodamines (**3a,b**) followed by amidation of 6'-carboxy isomer with Halo-tag amine **5**.

HPLC conditions (*Knauer GmbH,* Berlin, Germany): Smartline pump 1000 (2x) with 10 mL pump-head, UV detector 2500, column thermostat 4000 (25°C), mixing chamber, injection loop (20 µL); 6-port-3-channel switching valve; analytical column: *Eurospher-100 C18*, 5 µm, 250x4 mm, 1.2 mL/min; solvent A: water + 0.1% v/v trifluoroacetic acid (TFA); solvent B: MeCN + 0.1% v/v TFA. **3a (*540R*):** t_R = 11.0 min, **3b**: 12.0 min (A/B 90:10 → 0:100 + 0.1% in 25 min).

Compound **4** (***540R***-Halo): **HPLC:** t_R = 15.3 min (A/B 90:10 → 0:100 + 0.1% in 25 min, detected at 530 nm); **MS (ESI):** *m/z* (positive mode, %) = 660 (100) $[M+H]^+$. **HR-MS (ESI):** found 660.2814 $[M+H]^+$, M = $C_{37}H_{42}ClN_3O_6$ (659,2762), calcd. for $[M+H]^+$: 660.2835. **^{1}H NMR** (400 MHz, DMSO-*d*$_6$): *d* (ppm) = 1.17-1.47 (m, 6 H, CH_2), 1.65-1.72 (m, 6 H, $CH_2C\underline{H}_2CH_2Ar$ + CH_2), 1.95-2.04 (m, 2 H, CH_2), 2.40-2.59 (m, 4 H, $CH_2CH_2C\underline{H}_2Ar$, overlaps with solvent signal), 3.12-3.20 (m, 2 H, CH_2, overlaps with water signal), 3.30-3.45 (m, 6 H, CH_2, overlaps with water signal), 3.46-3.52 (m, 4 H, $C\underline{H}_2CH_2CH_2Ar$), 3.56-3.64 (m, 2 H, CH_2), 6.18 (s, 2 H, 4-H, 5-H) 6.31 (s, 2.00 H, 1-H, 8-H), 6.64 (br. s, 2 H, NH), 7.63 (d, $^4J_{H,H}$ = 1 Hz, 1.00 H, 7´-H), 8.01 (d, $^3J_{H,H}$ = 8.1 Hz, 1 H, 4´-H), 8.12 (dd, $^3J_{H,H}$ = 8.1 Hz, $^4J_{H,H}$ = 1 Hz, 1.00 H, 5´-H), 8.76 (br. s, CONH). **^{13}C NMR** (101 MHz, DMSO-*d*$_6$, extracted from HSQCAD): *d* (ppm) = 21.3, 22.5, 25.3, 25.5, 26.6, 29.2, 29.4, 32.4, 35.5, 39.8, 40.8, 45.8, 69.1, 70.0, 70.1, 98.1, 123.6, 125.8, 128.0, 129.3. **UV-Vis** (MeOH): λ_{max}= 540 nm (absorption), λ_{max} = 560 nm (emission, QY = 0.89, reference - Rh6G with QY = 0.9).

Supplementary References

1 Boyarskiy, V. P. *et al.* Photostable, Amino Reactive and Water-Soluble Fluorescent Labels Based on Sulfonated Rhodamine with a Rigidized Xanthene Fragment. *Chem. Eur. J.* **14**, 1784-1792 (2008).

2 Stagge, F., Mitronova, G. Y., Belov, V. N., Wurm, C. A. & Jakobs, S. Snap-, CLIP- and Halo-Tag Labelling of Budding Yeast Cells. *PLoS One* **8**, e78745 (2013).

3 Correa, I. *et al.* Substrates for Improved Live-Cell Fluorescence Labeling of SNAP-tag. *Curr. Pharm. Des.* **19**, 5414-5420 (2013).

4 Los, G. V. *et al.* HaloTag: A Novel Protein Labeling Technology for Cell Imaging and Protein Analysis. *ACS Chem. Biol.* **3**, 373-382 (2008).

5 <http://www.promegaconnections.com/tag/halotag> (Accessed: 10th March 2016).

6 Butkevich, A. N. *et al.* Fluorescent Rhodamines and Fluorogenic Carbopyronines for Super-Resolution STED Microscopy in Living Cells. *Angew. Chem. Int. Ed.* **55**, 3290-3294 (2016).

7 Butkevich, A. N. *et al.* Fluoreszierende Rhodamine und fluorogene Carbopyronine für die STED-Mikroskopie lebender Zellen. *Angew. Chem.* **55**, 3350-3355 (2016).
